# Effect of initial recurrence site on the prognosis of different tissue types of non-small cell lung cancer: a retrospective cohort study

**DOI:** 10.1186/s12957-023-03252-x

**Published:** 2023-11-21

**Authors:** Yanli Li, Lizhu Liu, Ruiming You, Qingwan Li, Zhaojuan Jiang, Hongjiang Pu, Zhenhui Li, Xiaobo Chen

**Affiliations:** 1 Department of Radiology, Yunnan Cancer Centre, the Third Affiliated Hospital of Kunming Medical University, Yunnan Cancer Hospital, Kunming, 650118 China; 2 Department of Oncology, Dazhou Central Hospital, Dazhou, 635000 Sichuan China; 3 First Department of Thoracic Surgery, Yunnan Cancer Centre, the Third Affiliated Hospital of Kunming Medical University, Yunnan Cancer Hospital, Kunming, 650118 China

**Keywords:** Non-small cell lung cancer, Site of initial recurrence, Post-recurrence survival, Prognostic

## Abstract

**Purpose:**

To explore the correlation between the initial recurrence site and survival after recurrence (PRS) in non-small cell lung cancer (NSCLC).

**Methods:**

We collected 588 stages I–III NSCLC patients with recurrence after radical resection in Yunnan Cancer Hospital from January 2013 to December 2018. We used Kaplan–Meier survival curves to compare PRS in patients with different site recurrences. The univariate and multivariate Cox proportional hazard models were used to analyze the impact of the initial recurrence site on PRS.

**Results:**

The recurrence site included the lung (*n* = 109), brain (*n* = 113), bone (*n* = 79), abdomen (*n* = 28), pleura (*n* = 24), lymph node (*n* = 81), and multisite (*n* = 154). In the total population, patients with multisite recurrence had substantially worse PRS (24.8 months, 95% confidence interval [CI]: 17.46–32.20) than that of patients without multiple sites recurrence (42.2 months, 95% *CI* 32.24–52.10) (*P* = 0.026). However, patients with lung recurrence had better RFS (63.1 months, 95% *CI* 51.13–74.00) than those who did not (31.0 months, 95% *CI* 25.10–36.96) (*P* < 0.001). In adenocarcinoma, patients with pleural recurrence had substantially worse PRS (21.3 months, 95% *CI* 15.07–27.46) than that of patients without pleural recurrence (46.9 months, 95% *CI* 35.07–58.80) (*P* = 0.031). Multivariate Cox proportional hazards regression analysis revealed that lung recurrence (*HR* 0.58, 95% *CI* 0.40–0.82; *P* = 0.003) was independent protective prognostic factor for PRS in the total population, while pleural recurrence (*HR* 2.18, 95% *CI* 1.14–4.17; *P* = 0.018) was independent adverse prognostic factors for PRS in adenocarcinoma patients.

**Conclusion:**

The initial recurrence site was associated with PRS in NSCLC patients. Identification of recurrence sites could guide the subsequent treatment.

**Supplementary Information:**

The online version contains supplementary material available at 10.1186/s12957-023-03252-x.

## Introduction

Lung cancer has a high incidence and fatality rate globally [1]. Approximately, 40% of stages I–III non-small cell lung cancer (NSCLC) patients often recur after radical resection [2–5]. The prognosis following recurrence was poor, and less than 2 years have been recorded as survival following a recurrence of primary lung cancer [4–8]. Studies have also shown that postoperative chemotherapy and radiotherapy can improve the PRS of patients [8, 9]. However post-recurrence survival (PRS) varied significantly among different patients. The PRS was negatively impacted by males [4, 6, 7], older age [4, 7, 10], poor motor status [5], abdominal and bone metastases [7], and poor differentiation [6, 10, 11]. Identifying risk factors affecting survival after relapse could help clinicians to make the subsequent treatment regimen. A lot of studies have explored or predicted prognostic factors for relapse-free survival in non-small cell lung cancer [12–14]. However, little is known about the factors affecting PRS in NSCLC patients, and further studies are needed.

Few studies have explored the association of recurrence sites with PRS in NSCLC patients, particularly adenocarcinoma patients. Some studies show that patients with local recurrence, as opposed to distant recurrence, have a better PRS [13, 15]. However, in several studies, distant recurrence did not impact PRS [5, 6, 16]. Besides, some studies show that poor PRS was seen in patients with bone metastases [7, 9, 11], liver metastases [6, 8], lung metastases [4], and abdominal organ metastases [7]. The survival time of liver metastasis was 7.8 months [7]. Median survival with brain metastases was 7–10 months [17]. The more metastatic organs, the shorter the survival time, but there was no significant difference in the survival time of patients with three or four or more metastatic organs [17]. Inaccurate identification of the initial recurrence site is the reason for the poor prognosis of patients after recurrence. However, there is limited research on the effect of each recurrence site on postoperative prognosis. Furthermore, there is no consensus on which recurrence site has a poorer PRS when NSCLC or adenocarcinoma first recurs.

Hence, this study aimed to explore the correlation between the initial recurrence site and PRS in NSCLC patients; to find out which organ metastasis of non-small cell lung cancer has the worst prognosis, especially adenocarcinoma; and to guide clinical decision-making and improve patient prognosis.

## Patients and methods

### Ethics statement

The study protocol (KY2019141) was reviewed and approved by the Institutional Review Board of the Yunnan Cancer Hospital. It was carried out following the fundamental principles of the Declaration of Helsinki. Informed consent was waived due to the retrospective nature of the study.

### Patients

**Fig. 1:**
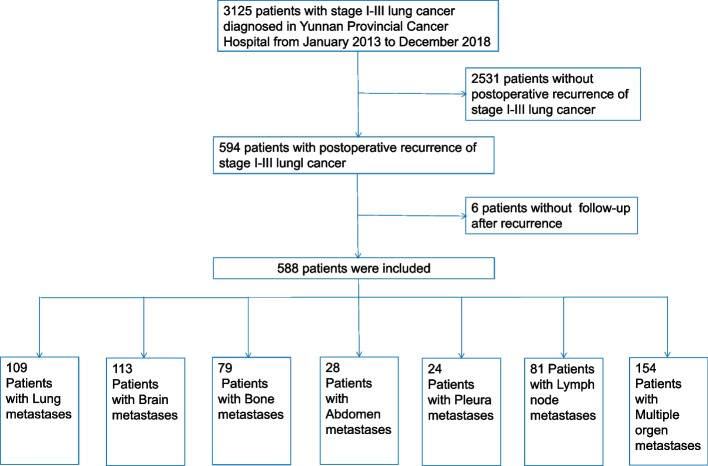
Inclusion and exclusion flow charts

From January 2013 to December 2018, this retrospective study collected consecutively 3125 patients with pathologically confirmed stages I–III primary lung cancer in Yunnan Cancer Hospital after radical resection of lung cancer. Among them, 588 patients with recurrence were included in the analysis. A total of 2531 patients with recurrence and 6 patients who were lost to follow-up were excluded (Fig. 1).

### Observation following surgery and recurrence

Chest and abdomen CT and blood tumor markers were routinely performed every 3 to 6 months within 3 years after surgery, and checkups were performed every 6 to 12 months for 4–5 years after surgery. Imaging examinations were carried out following symptoms when symptoms emerged during follow-up: positron emission tomography (PET)-CT or bone scan for bone pain, magnetic resonance imaging (MRI) of the head for headaches, contrast-enhanced CT or MRI, abdominal ultrasound, or gastrointestinal endoscopy for abdominal pain. After the identification of recurrence, any further systemic treatment was at the discretion of the multidisciplinary team.

### Initial recurrence organs classification and word meanings

The initial recurrence organs were divided into the following seven subgroups: the (i) lung, (ii) brain, (iii) bone, (iv) abdomen (liver and adrenal glands), (v) pleural, (vi) lymph node, and (vii) multisite (two or more organs). PRS in patients with relapsing sites was analyzed. PRS was defined as the time to all-cause death from the first time there was evidence of relapse to the last observation period in an event-free review patient.

### Statistical analysis

For continuous variables, the *t*-test was employed. For categorical variables, the Fisher exact test was employed. Both univariate and multivariate analyses for PRS were conducted using the Cox proportional hazard model. The variables included age, sex, BMI, surgical modality, degree of differentiation, pathological stage of AJCC, vascular invasion, bronchial invasion, pleural invasion, recurrent sites (lung, brain, bone, abdomen, pleura, lymph nodes, multisite), two recurrent sites, three or more relapsed sites, adjuvant chemotherapy, radiotherapy, and targeted therapy. For variables in the univariate analysis with *P* < 0.05, multivariate analysis was carried out. The Kaplan–Meier method was employed for calculating PRS. The log-rank test was performed to compare groups. *P* < 0.05 is regarded as significant in statistical terms. R4.2.2 (R project) was used for statistical analysis.

## Results

In the end, 588 patients were included in the study following the inclusion and exclusion criteria. The median age of patients was 58.00 (33.00–85.00), and the median BMI was 22.65 (6.38–68.89) (Table 1). There were 109 cases (18.54%) of lung recurrence, 113 cases (19.22%) of brain recurrence, 79 cases (13.44%) of bone recurrence, 28 cases (4.76%) of abdominal recurrence, 24 cases (4.08%) of pleural recurrence, 154 cases (26.19%) of multisite recurrence, 81 cases (13.78%) of lymph node recurrence, and 28 cases (4.76%) of abdominal recurrence. There were 14 cases of liver metastasis and 14 cases of adrenal metastasis among patients with recurrence of abdominal organs. The median PRS in the total population was 23.75 (range 0.03–104.97) months. The PRS for adenocarcinoma was 25.05 (0.07–104.97) months (Table 1).

**Table 1:** Clinicopathological characteristics of patients

Characteristic	All patients (*n* = 588)	Adenocarcinoma patients (*n* = 427)	Squamous carcinoma patients (*n* = 116)	Other patients^4^ (*n* = 45)	*p*-value^6^
Age (years) Mean (SD) Median (IQR)	58.66 (9.32) 58.00 (33.00–85.00)	57.85 (9.33) 57.00 (33.00–85.00)	61.49 (8.38) 62.00 (42.00–83.00)	59.07 (10.28) 59.00 (38.00–84.00)	< 0.001
BMI^1^ (kg/m^2^) Mean (SD) Median (IQR)	23.06 (3.71) 22.65 (6.38–68.89)	23.10 (3.91) 22.83 (6.38–68.89)	22.86 (3.11) 22.40 (14.86–31.49)	23.14 (3.27) 22.38 (18.07–32.32)	0.816
PRS (M) Mean (SD) Median (IQR)	28.78 (22.03) 23.75 (0.03–104.97)	30.01 (22.52) 25.05 (0.07–104.97)	25.91 (20.38) 21.00 (0.03–102.40)	24.62 (20.61) 17.82 (2.40–102.80)	0.089
Sex					< 0.001
Male	359 (61.05%)	213 (49.88%)	113 (97.41%)	33 (73.33%)	
Female	229 (38.95%)	214 (50.12%)	3 (2.59%)	12 (26.67%)	
Surgical mode					< 0.001
Lobectomy	508 (86.39%)	367 (85.95%)	101 (87.07%)	40 (88.89%)	
Segmentectomy	6 (1.02%)	6 (1.41%)	0 (0.00%)	0 (0.00%)	
Wedge resection	54 (9.18%)	50 (11.71%)	2 (1.72%)	2 (4.44%)	
Total pneumonectomy	20 (3.40%)	4 (0.94%)	13 (11.21%)	3 (6.67%)	
Tumor differentiation					< 0.001
Unknown	377 (64.12%)	329 (77.05%)	16 (13.79%)	32 (71.11%)	
Medium differentiation	98 (16.67%)	41 (9.60%)	55 (47.41%)	2 (4.44%)	
Low differentiation	98 (16.67%)	48 (11.24%)	44 (37.93%)	6 (13.33%)	
Undifferentiation	10 (1.70%)	4 (0.94%)	1 (0.86%)	5 (11.11%)	
Medium–low differentiation	5 (0.85%)	5 (1.17%)	0 (0.00%)	0 (0.00%)	
Vascular cancer thrombus					0.820
Yes	23 (3.91%)	17 (3.98%)	5 (4.31%)	1 (2.22%)	
No	565 (96.09%)	410 (96.02%)	111 (95.69%)	44 (97.78%)	
Bronchial stump					0.600
Yes	38 (6.46%)	25 (5.85%)	9 (7.76%)	4 (8.89%)	
No	550 (93.54%)	402 (94.15%)	107 (92.24%)	41 (91.11%)	
Pleural invasion					0.158
Yes	109 (18.83%)	72 (17.06%)	28 (25.00%)	9 (20.00%)	
No	470 (81.17%)	350 (82.94%)	84 (75.00%)	36 (80.00%)	
AJCC^1^ 8th ed. stage					0.225
AICC < = II stage	308 (52.38%)	233 (54.57%)	54 (46.55%)	21 (46.67%)	
AICC > II stage	280 (47.62%)	194 (45.43%)	62 (53.45%)	24 (53.33%)	
Adjuvant chemotherapy					0.019
Yes	479 (81.46%)	336 (78.69%)	103 (88.79%)	40 (88.89%)	
No	109 (18.54%)	91 (21.31%)	13 (11.21%)	5 (11.11%)	
Adjuvant radiation therapy					0.002
Yes	174 (29.59%)	111 (26.00%)	41 (35.34%)	22 (48.89%)	
No	414 (70.41%)	316 (74.00%)	75 (64.66%)	23 (51.11%)	
Postoperative targeted therapy					0.142
Yes	45 (7.65%)	38 (8.90%)	4 (3.45%)	3 (6.67%)	
No	543 (92.35%)	389 (91.10%)	112 (96.55%)	42 (93.33%)	
Two sites					0.935
Yes	81 (13.78%)	58 (13.58%)	16 (13.79%)	7 (15.56%)	
No	507 (86.22%)	369 (86.42%)	100 (86.21%)	38 (84.44%)	
Three or more recurrence sites					0.514
Yes	64 (10.88%)	46 (10.77%)	15 (12.93%)	3 (6.67%)	
No	524 (89.12%)	381 (89.23%)	101 (87.07%)	42 (93.33%)	
Lung recurrence					0.030
Yes	109 (18.54%)	90 (21.08%)	15 (12.93%)	4 (8.89%)	
No	479 (81.46%)	337 (78.92%)	101 (87.07%)	41 (91.11%)	
Brain recurrence					0.012
Yes	113 (19.22%)	93 (21.78%)	11 (9.48%)	9 (20.00%)	
No	475 (80.78%)	334 (78.22%)	105 (90.52%)	36 (80.00%)	
Bone recurrence					0.172
Yes	79 (13.44%)	56 (13.11%)	13 (11.21%)	10 (22.22%)	
No	509 (86.56%)	371 (86.89%)	103 (88.79%)	35 (77.78%)	
Abdominal organs^2^ recurrence					0.023
Yes	28 (4.76%)	14 (3.28%)	10 (8.62%)	4 (8.89%)	
No	560 (95.24%)	413 (96.72%)	106 (91.38%)	41 (91.11%)	
Pleural recurrence					0.479
Yes	24 (4.08%)	15 (3.51%)	6 (5.17%)	3 (6.67%)	
No	564 (95.92%)	412 (96.49%)	110 (94.83%)	42 (93.33%)	
Lymph node^5^ recurrence					< 0.001
Yes	81 (13.78%)	46 (10.77%)	30 (25.86%)	5 (11.11%)	
No	507 (86.22%)	381 (89.23%)	86 (74.14%)	40 (88.89%)	
Multisite^3^ recurrence					0.819
Yes	154 (26.19%)	113 (26.46%)	31 (26.72%)	10 (22.22%)	
No	434 (73.81%)	314 (73.54%)	85 (73.28%)	35 (77.78%)	

^1^Abbreviations: *BMI*, body mass index; *AJCC*, American Joint Committee on Cancer. ^2^Abdominal organs (liver + adrenal). ^3^Multisite (two or more organs). ^4^Other patients (neuroendocrine carcinoma, adenosquamous cell carcinoma, carcinoid). ^5^Lymph node (hilar, supraclavicular, and thoracic lymph nodes). ^6^*P*-value, using Pearson’s chi-squared test. Wilcoxon rank-sum test. Fisher’s exact test

In the total population, in comparison to patients without multisite recurrence (42.2 months, 95% *CI* 32.24–52.10), patients with multisite recurrence (24.8 months, 95% *CI* 17.46–32.20) (*P* = 0.026) had substantially worse PRS (Figure S1G). However, patients who had lung recurrence (63.1 months, 95% *CI* 51.13–74.00) had better PRS than patients who did not (31.0 months, 95% 25.10–36.96) (*P* < 0.001) (Figure S1A). Patients with or without brain, bone, abdominal, pleural, and lymph node recurrence were not statistically significant (Figure S1).

**Fig. 2:**
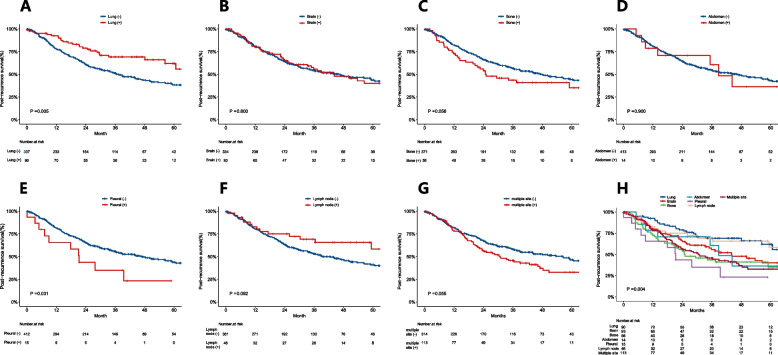
The PRS of patients with pleural recurrence was worse than that of patients without pleural recurrence (**E**), and lung recurrence is the opposite (**A**) in adenocarcinoma. There were no significant differences in the PRS of patients with or without recurrence at other sites (**B**–**C** and **F**–**G**). Among all recurrence sites, pleural recurrence has the worst prognosis (**H**). PRS, post-recurrence survival

In adenocarcinoma, in comparison to patients without pleural recurrence (46.9 months, 95% *CI* 35.07–58.80), patients with pleural recurrence (21.3 months, 95% *CI* 15.07–27.46) (*P* = 0.031) had substantially worse PRS (Fig. 2E). However, patients who did lung recurrence (63.1 months, 95% *CI* 54.36–71.78) had better RFS than those who did not (37.3 months, 95% *CI* 29.0–45.60) (*P* = 0.005) (Fig. 2A). Patients with or without brain, bone, abdomen, lymph nodes, and multisite recurrence were not statistically significant (Fig. 2). In Fig. 2H, patients with pleural recurrence (21.3 months, 95% *CI* 15.07–27.46) (*P* = 0.004) had the worst PRS.

In squamous cell carcinoma, in comparison to patients without multisite recurrence (30.0 months, 95% *CI* 21.84–38.16), patients with multisite recurrence (17.8 months, 95% *CI* 11.19–24.41; *P* = 0.038) had substantially worse PRS (Figure S2G). Patients with or without lung, brain, bone, abdomen, lymph nodes, and pleural recurrence were not statistically significant (Figure S2).

In multivariate analysis of the general population, lung recurrence was a protected factor for PRS (*HR* 0.58, 95% *CI* 0.40–0.82; *P* = 0.003), and female, BMI (≥ 24), vascular invasion, AICC > II stage, and three or more recurrent sites were poor prognostic factors for PRS (Table S1).

In multivariate analysis of the adenocarcinoma, lung recurrence was a protective factor for PRS (*HR* 0.62, 95% *CI* 0.41–0.95; *P* = 0.027), and pleural recurrence (*HR* 2.18, 95% *CI* 1.14–4.17; *P* = 0.018), vascular invasion, AICC > II stage, and three or more recurrent sites were poor prognostic factors for PRS (Table 2). In multivariate analysis of the squamous cell carcinoma, older age (≥ 60 years) and three or more recurrence sites were poor prognostic factors for PRS (Table S2).

**Table 2:** Univariate and multivariate analysis of recurrence survival for adenocarcinoma

Post-recurrence survival variable	Univariate HR^1^ (95% *CI*^1^)	*p*-value^5^	Multivariate HR^1^ (95% *CI*^1^)	*p*-value^5^
Sex				
Male	1.0 (reference)			
Female	0.88 (0.66, 1.16)	0.363		
Age group				
< 60	1.0 (reference)			
≥ 60	0.98 (0.73, 1.30)	0.884		
BMI^1^ group				
< 24	1.0 (reference)			
≥ 24	0.74 (0.55, 1.00)	0.052		
Surgical mode				
Lobectomy	1.0 (reference)			
Segmentectomy	0.29 (0.04, 2.05)	0.213		
Wedge resection	1.44 (0.96, 2.16)	0.081		
Total pneumonectomy	1.29 (0.32, 5.20)	0.722		
Tumor differentiation				
Unknown	1.0 (reference)			
Medium differentiation	0.85 (0.52, 1.39)	0.522		
Low differentiation	1.19 (0.78, 1.80)	0.420		
Undifferentiation	0.50 (0.07, 3.57)	0.488		
Medium–low differentiation	1.88 (0.60, 5.89)	0.281		
Vascular cancer thrombus				
No	1.0 (reference)		1.0 (reference)	
Yes	1.93 (1.08, 3.47)	0.027	2.00 (1.11, 3.61)	0.021
Bronchial stump				
No	1.0 (reference)			
Yes	1.62 (0.98, 2.67)	0.058		
Pleural invasion				
No	1.0 (reference)			
Yes	0.85 (0.56, 1.28)	0.427		
AJCC^1^ 8th ed. stage				
AICC < = II stage	1.0 (reference)		1.0 (reference)	
AICC > II stage	1.78 (1.34, 2.37)	< 0.001	1.70 (1.27, 2.27)	< 0.001
Adjuvant chemotherapy				
No	1.0 (reference)			
Yes	0.87 (0.61, 1.22)	0.415		
Adjuvant radiation therapy				
No	1.0 (reference)			
Yes	0.89 (0.65, 1.22)	0.474		
Postoperative targeted therapy				
No	1.0 (reference)			
Yes	0.97 (0.61, 1.54)	0.891		
Two sites				
No	1.0 (reference)			
Yes	0.96 (0.63, 1.45)	0.839		
Three or more recurrence sites				
No	1.0 (reference)		1.0 (reference)	
Yes	1.80 (1.22, 2.66)	0.003	1.60 (1.07, 2.39)	0.022
Lung recurrence				
No	1.0 (reference)		1.0 (reference)	
Yes	0.56 (0.37, 0.84)	0.005	0.62 (0.41, 0.95)	0.027
Brain recurrence				
No	1.0 (reference)			
Yes	0.96 (0.68, 1.35)	0.804		
Bone recurrence				
No	1.0 (reference)			
Yes	1.44 (0.99, 2.09)	0.058		
Abdominal organs^2^ recurrence				
No	1.0 (reference)			
Yes	1.05 (0.49, 2.24)	0.896		
Pleural recurrence				
No	1.0 (reference)		1.0 (reference)	
Yes	1.99 (1.05, 3.77)	0.034	2.18 (1.14, 4.17)	0.018
Lymph node^4^ recurrence				
No	1.0 (reference)			
Yes	0.64 (0.38, 1.08)	0.094		
Multisite^3^ recurrence				
No	1.0 (reference)			
Yes	1.35 (0.99, 1.83)	0.057		

^1^Abbreviations: *BMI*, body mass index; *AJCC*, American Joint Committee on Cancer; *CI*, confidence interval; *HR*, hazard ratio. ^2^Abdominal organs (liver + adrenal). ^3^Multisite (two or more organs). ^4^Lymph node (hilar, supraclavicular, and thoracic lymph nodes). ^5^*P*-value, using Pearson’s chi-squared test. Wilcoxon rank-sum test. Fisher’s exact test

## Discussion

This study found that lung recurrence was an independent prognostic factor for PRS in the general population. Still, brain recurrence, bone recurrence, abdominal recurrence, pleural recurrence, lymph node recurrence, and multiple site recurrence did not affect PRS. In adenocarcinoma, lung and pleural recurrence were independent prognostic factors for PRS. In contrast, brain recurrence, bone recurrence, abdominal recurrence, lymph node recurrence, and multiple site recurrence did not affect PRS. It was not found which single-organ recurrence was an independent prognostic factor for PRS in squamous cell carcinoma.

Previous studies have reported that recurrence at two or more sites does not affect the prognosis of lung cancer [7]. However, in this study, we divided them into two subgroups: two recurrence sites and three or more recurrence sites. Therefore, multivariate analysis showed three or more recurrence sites with statistical significance and poor prognosis. In comparison, two recurrence sites were not statistically significant in the general population, adenocarcinoma, and squamous cell carcinoma.

Different studies have found different prognoses of PRS at various recurrence sites, such as liver recurrence. Some studies have shown that RFS in patients with abdominal organ recurrence is significantly worse and frequently recurrent [7, 18]. Additionally, it has been reported that the prognosis was poor when the liver was the first abdominal organ to recur following lung cancer resection [5, 8]; according to earlier research, patients with advanced lung cancer liver metastases also had a poor prognosis due to chemotherapy [19], TKI therapy [7, 18], immunological tolerance [20], and quicker tumor growth [21]. Nevertheless, some studies have shown that liver metastases do not influence PRS [7, 9, 11].

Different investigations have found other effects of lung recurrence on PRS prognosis. One study showed that patients with lung recurrence [4, 8] had better PRS, possibly because the lung recurrence was less malignant, which was also confirmed in our study. Moreover, radiotherapy and metastasectomy at lung recurrence sites may improve PRS in patients with lung recurrence [22, 23]. However, some research shows lung recurrence has little impact on PRS [6, 9, 11].

Some studies reported that pleural recurrence has nothing to do with PRS among lung cancer patients [7]. However, they did not do a subgroup analysis. Our study found through subgroup analysis that pleural recurrence was not statistically significant in the general population and squamous cell carcinoma in our study. Our finding was consistent with previous findings [7]. However, in adenocarcinoma, pleural recurrence patients have a poorer prognosis, possibly because there are few studies on pleural metastasis in lung cancer. A larger cohort may be needed to investigate further prognostic factors affecting pulmonary adenocarcinoma pleural recurrence to improve PRS in patients with adenocarcinoma pleural recurrence.

This study belongs to a large cohort study. For the first time, the impact of initial recurrence organs on lung cancer PRS was divided into the total population, adenocarcinoma, and squamous cell carcinoma to study separately, and the initial recurrence organs were divided into seven subgroups. There were a few limitations to this study. First, because this study was retrospective and only involved one institution, selection bias could not be ruled out; second, the method of postoperative monitoring was different for each doctor. Third, this study included fewer squamous cell carcinoma patients (*n* = 116), and the subsequent research could consist of more squamous cell carcinoma patients to analyze which initial recurrence site had the most significant effect on PRS in their population.

In conclusion, the initial recurrence site was associated with PRS in NSCLC patients. Identification of recurrence sites could guide the subsequent treatment. In adenocarcinoma, patients with pleural recurrence have the worst prognosis, which should be followed up as soon as possible, and early intervention treatment to improve the prognosis of patients. Patients of squamous cell carcinoma should pay more attention to multisite recurrence.

### Supplementary Information

**Additional file 1: Figure S1.** The PRS of patients with lung and multiple site recurrence was worse than that of patients without lung and multiple site recurrence(G) and lung recurrence is the opposite in adenocarcinoma (A) in the total population. There were no significant differences in the PRS of patients with or without recurrence at other sites (B-F). Among all recurrence sites, lung recurrence has the best prognosis(H).PRS: post-recurrence survival.

**Additional file 2: Figure S2.** The PRS of patients with multiple site recurrence was worse than that of patients without multiple site recurrence in squamous cell carcinoma (G). There were no significant differences in the PRS of patients with or without recurrence at other sites (A-F, H).PRS: post-recurrence survival.

**Additional file 3: Table S1.** Univariate and multivariate analyses of post-recurrence survival in the general population.

**Additional file 4: Table S2.** Univariate and multivariate analysis of recurrence survival in squamous cell carcinoma.

## Data Availability

The datasets generated and/or analyzed during the current study are not publicly available because they are related to patients but are available from the corresponding author on request.

## Abbreviations

CI: Confidence interval
CT: Computed tomography
MRI: Magnetic resonance imaging
PET: Positron emission tomography
HR: Hazard ratio
MRI: Magnetic resonance imaging
NSCLC: Non-small cell lung cancer
PRS: Post-recurrence survival
RFS: Relapse-free survival
TKI: Tyrosine kinase inhibitor
BMI: Body mass index
