# Supplementary material for: Effect of initial recurrence site on the prognosis of different tissue types of non-small cell lung cancer: a retrospective cohort study
Source: World J Surg Oncol. 2023 Nov 21;21:360. doi: 10.1186/s12957-023-03252-x (PMC10662500; doi:10.1186/s12957-023-03252-x)
Supplement: Supplementary file 1 — Additional file 1: Figure S1. The PRS of patients with lung and multiple site recurrence was worse than that of patients without lung and multiple site recurrence(G) and lung recurrence is the opposite in adenocarcinoma (A) in the total population. There were no significant differences in the PRS of patients with or without recurrence at other sites (B-F). Among all recurrence sites, lung recurrence has the best prognosis(H).PRS: post-recurrence survival. [file 12957_2023_3252_MOESM1_ESM.pdf]

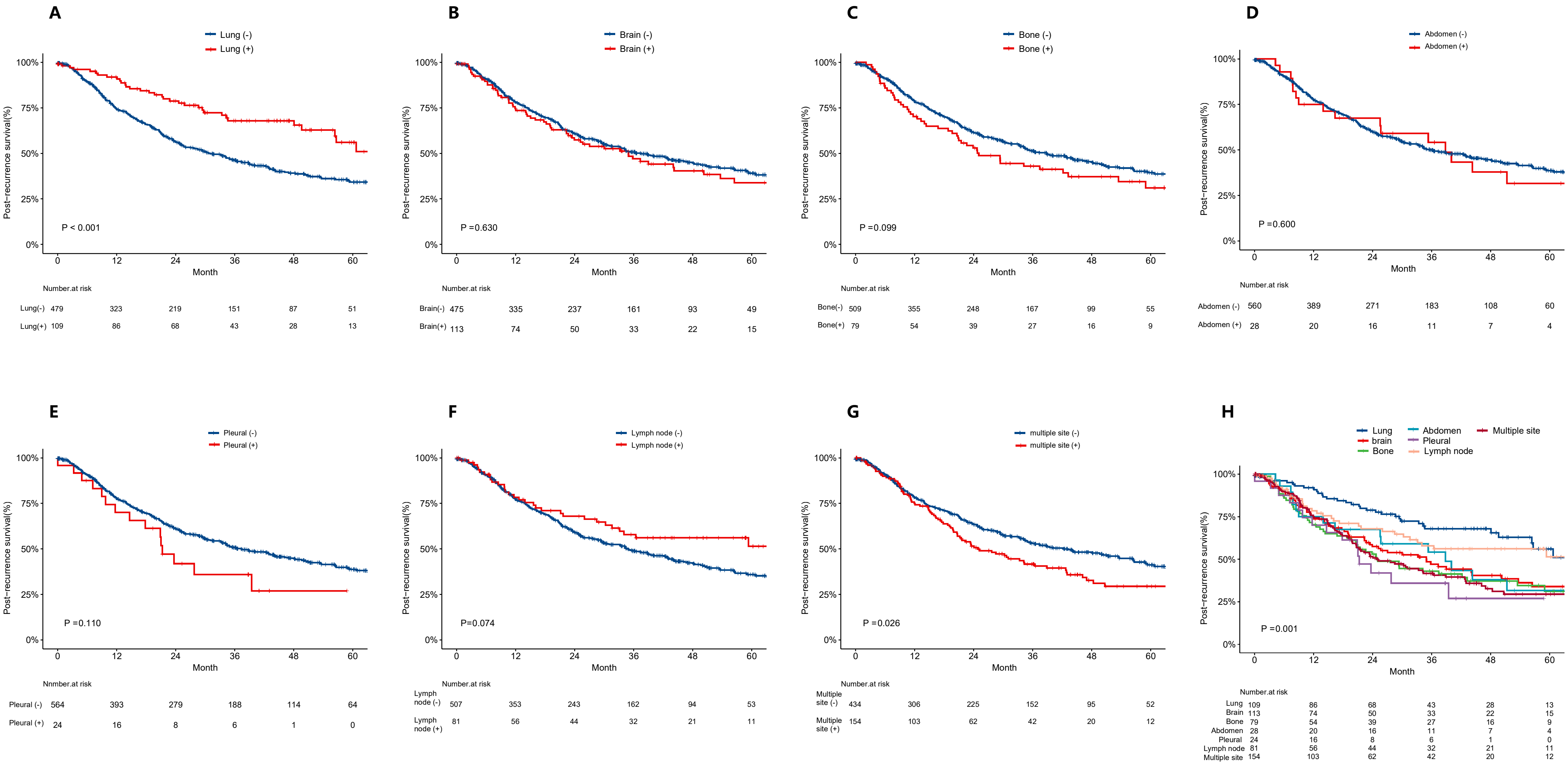

Figure S1: The PRS of patients with lung and multiple site recurrence was worse than that of patients without lung and multiple site recurrence(G) and lung recurrence is the opposite in adenocarcinoma (A) in the total population. There were no significant differences in the PRS of patients with or without recurrence at other sites (B-F).Among all recurrence sites, lung recurrence has the best prognosis(H) .PRS: post-recurrence survival.
