# Supplementary material for: Effect of initial recurrence site on the prognosis of different tissue types of non-small cell lung cancer: a retrospective cohort study
Source: World J Surg Oncol. 2023 Nov 21;21:360. doi: 10.1186/s12957-023-03252-x (PMC10662500; doi:10.1186/s12957-023-03252-x)
Supplement: Supplementary file 2 — Additional file 2: Figure S2. The PRS of patients with multiple site recurrence was worse than that of patients without multiple site recurrence in squamous cell carcinoma (G). There were no significant differences in the PRS of patients with or without recurrence at other sites (A-F, H).PRS: post-recurrence survival. [file 12957_2023_3252_MOESM2_ESM.pdf]

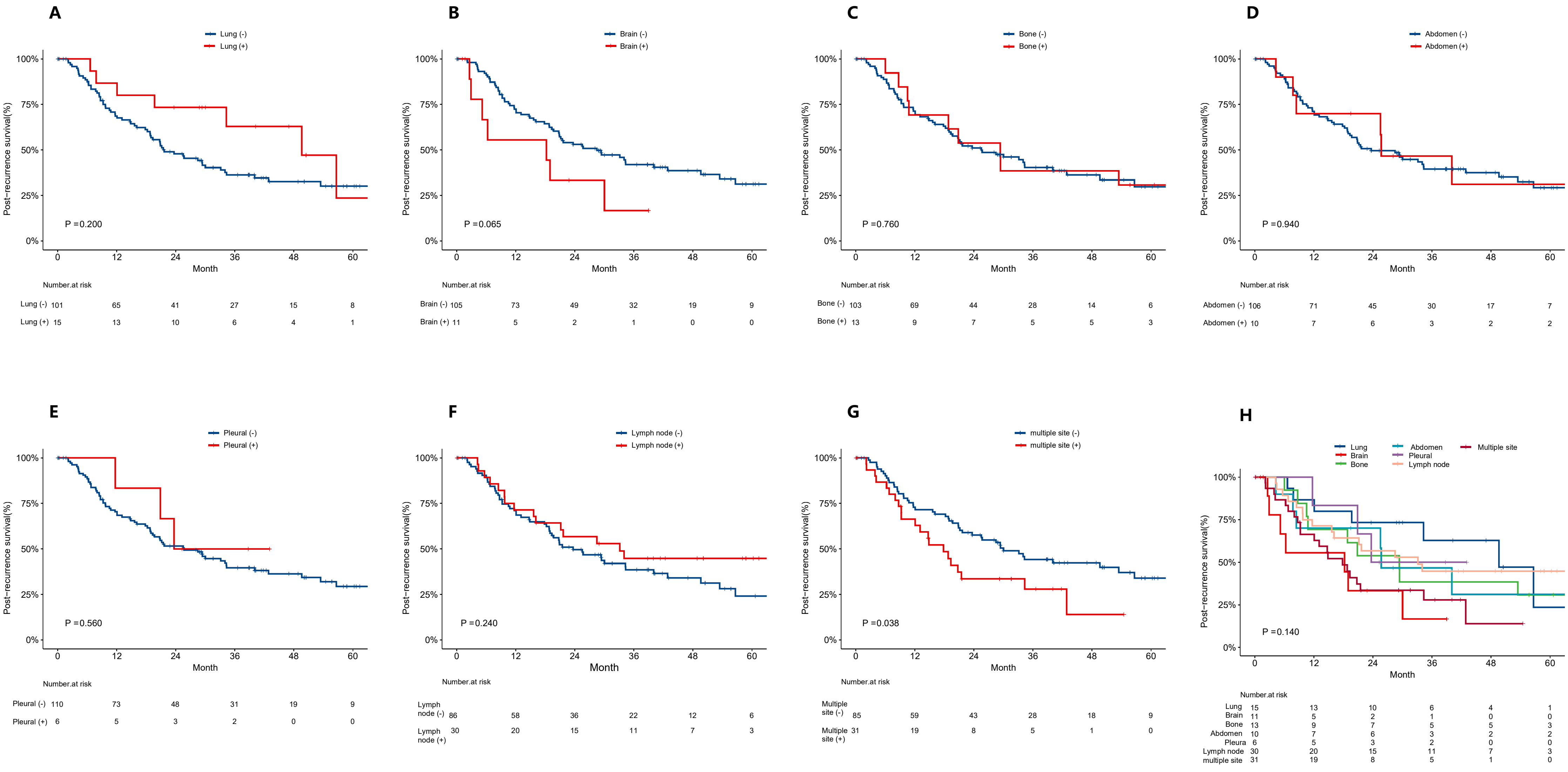

Figure S2: The PRS of patients with multiple site recurrence was worse than that of patients without multiple site recurrence in squamous cell carcinoma (G). There were no significant differences in the PRS of patients with or without recurrence at other sites (A-F,H).PRS: post-recurrence survival.
