# Supplementary material for: Effect of initial recurrence site on the prognosis of different tissue types of non-small cell lung cancer: a retrospective cohort study
Source: World J Surg Oncol. 2023 Nov 21;21:360. doi: 10.1186/s12957-023-03252-x (PMC10662500; doi:10.1186/s12957-023-03252-x)
Supplement: Supplementary file 3 — Additional file 3: Table S1. Univariate and multivariate analyses of post-recurrence survival in the general population. [file 12957_2023_3252_MOESM3_ESM.docx]

Table S1. Univariate and multivariate analyses of post-recurrence survival in the total population

| Post-recurrence survival variable | Univariate  HR^1^ (95% CI^1^) | *P*-value^5^ | Multivariate  HR^1^ (95% CI^1^) | *P*-value^5^ |
| --- | --- | --- | --- | --- |
| Sex |  |  |  |  |
| Male | 1.0(reference) |  | 1.0(reference) |  |
| Female | 0.73(0.57,0.93) | 0.012 | 0.74(0.58,0.95) | 0.018 |
| Age group |  |  |  |  |
| <60 | 1.0(reference) |  |  |  |
| ≥60 | 1.00(0.79,1.25) | 0.979 |  |  |
| BMI^1^ group |  |  |  |  |
| <24 | 1.0(reference) |  | 1.0(reference) |  |
| ≥24 | 0.75(0.59,0.96) | 0.021 | 0.72(0.56,0.92) | 0.009 |
| Surgical mode |  |  |  |  |
| Lobectomy | 1.0(reference) |  |  |  |
| Segmentectomy | 0.25(0.03,1.76) | 0.163 |  |  |
| Wedge resection | 1.28(0.88,1.87) | 0.200 |  |  |
| Total pneumonectomy | 1.49(0.85,2.60) | 0.163 |  |  |
| Tumour differentiation |  |  |  |  |
| Unknown | 1.0(reference) |  |  |  |
| Medium differentiation | 1.20(0.88,1.62) | 0.248 |  |  |
| Low differentiation | 1.23(0.91,1.66) | 0.184 |  |  |
| Undifferentiation | 0.88(0.32,2.36) | 0.793 |  |  |
| Medium-Low differentiation | 1.73(0.55,5.43) | 0.344 |  |  |
| Vascular cancer thrombus |  |  |  |  |
| No | 1.0(reference) |  | 1.0(reference) |  |
| Yes | 1.68(1.03,2.74) | 0.039 | 1.68(1.03,2.76) | 0.0400 |
| Bronchial stump |  |  |  |  |
| No | 1.0(reference) |  | 1.0(reference) |  |
| Yes | 1.49(1.01,2.20) | 0.046 | 1.16(0.78,1.73) | 0.467 |
| Pleural invasion |  |  |  |  |
| No | 1.0(reference) |  |  |  |
| Yes | 1.00(0.74,1.35) | 0.983 |  |  |
| AJCC^1^ 8th ed. stage |  |  |  |  |
| AICC<=II stage | 1.0(reference) |  | 1.0(reference) |  |
| AICC>II stage | 1.62(1.29,2.04) | <0.001 | 1.5(1.84,1.9) | <0.001 |
| Adjuvant chemotherapy |  |  |  |  |
| No | 1.0(reference) |  |  |  |
| Yes | 0.94(0.69,1.27) | 0.670 |  |  |
| Adjuvant radiation therapy |  |  |  |  |
| No | 1.0(reference) |  |  |  |
| Yes | 1.02(0.80,1.30) | 0.869 |  |  |
| Postoperative targeted therapy |  |  |  |  |
| No | 1.0(reference) |  |  |  |
| Yes | 0.79(0.52,1.21) | 0.286 |  |  |
| Two site |  |  |  |  |
| No | 1.0(reference) |  |  |  |
| Yes | 0.99(0.71,1.37) | 0.940 |  |  |
| Three or more recurrence sites |  |  |  |  |
| No | 1.0(reference) |  | 1.0(reference) |  |
| Yes | 1.76(1.27,2.43) | <0.001 | 1.56(1.02,2.40) | 0.039 |
| Lung recurrence |  |  |  |  |
| No | 1.0(reference) |  | 1.0(reference) |  |
| Yes | 0.53(0.37,0.75) | <0.001 | 0.58(0.4,0.82) | 0.003 |
| Brain recurrence |  |  |  |  |
| No | 1.0(reference) |  |  |  |
| Yes | 1.07(0.80,1.44) | 0.626 |  |  |
| Bone recurrence |  |  |  |  |
| No | 1.0(reference) |  |  |  |
| Yes | 1.29(0.95,1.76) | 0.100 |  |  |
| Abdominal organs^2^ recurrence |  |  |  |  |
| No | 1.0(reference) |  |  |  |
| Yes | 1.14(0.70,1.86) | 0.604 |  |  |
| Pleural recurrence |  |  |  |  |
| No | 1.0(reference) |  |  |  |
| Yes | 1.53(0.91,2.58) | 0.108 |  |  |
| Lymph node^4^ recurrence |  |  |  |  |
| No | 1.0(reference) |  |  |  |
| Yes | 0.72(0.50,1.03) | 0.075 |  |  |
| Multisite^3^ recurrence |  |  |  |  |
| No | 1.0(reference) |  | 1.0(reference) |  |
| Yes | 1.33(1.03,1.71) | 0.026 | 1.00(0.72,1.41) | 0.975 |

Note：1. Abbreviations: BMI, Body Mass Index ; AJCC, American Joint Committee on Cancer ; CI, Confidence interval;HR, Hazard ratio

2. Abdominal organs (liver+Adrenal)

3. Multisite (2 or more organs)

4. Lymph node (hilar, supraclavicular and thoracic lymph nodes)

5. *P* value, using Pearson's Chi-squared test; Wilcoxon rank sum test; Fisher's exact test
