# Supplementary material for: Effect of initial recurrence site on the prognosis of different tissue types of non-small cell lung cancer: a retrospective cohort study
Source: World J Surg Oncol. 2023 Nov 21;21:360. doi: 10.1186/s12957-023-03252-x (PMC10662500; doi:10.1186/s12957-023-03252-x)
Supplement: Supplementary file 4 — Additional file 4: Table S2. Univariate and multivariate analysis of recurrence survival in squamous cell carcinoma. [file 12957_2023_3252_MOESM4_ESM.docx]

## Table S2.Univariate and multivariate analysis of recurrence survival in squamous cell carcinoma

| Post-recurrence survival variable | Univariate  HR^1^ (95% CI^1^) | *P*-value^5^ | Multivariate  HR^1^ (95% CI^1^) | *P*-value^5^ |
| --- | --- | --- | --- | --- |
| Sex |  |  |  |  |
| Male | 1.0(reference) |  |  |  |
| Female | 0.51 (0.07, 3.66) | 0.501 |  |  |
| AGE group |  |  |  |  |
| <60 | 1.0(reference) |  | 1.0(reference) |  |
| ≥60 | 1.68 (1.04, 2.73) | 0.036 | 1.63(1.00,2.65) | 0.048 |
| BMI^1^ group |  |  |  |  |
| <24 | 1.0(reference) |  |  |  |
| ≥24 | 0.89 (0.54, 1.44) | 0.628 |  |  |
| Surgical mode |  |  |  |  |
| Lobectomy | 1.0(reference) |  |  |  |
| Wedge resection | 1.75 (0.42, 7.20) | 0.440 |  |  |
| Total pneumonectomy | 1.46 (0.72, 2.97) | 0.289 |  |  |
| Tumour differentiation |  |  |  |  |
| Unknown | 1.0(reference) |  |  |  |
| Medium differentiation | 1.09 (0.54, 2.20) | 0.814 |  |  |
| Low differentiation | 0.78 (0.37, 1.64) | 0.509 |  |  |
| Undifferentiation | 0.00 (0.00, Inf) | 0.996 |  |  |
| Vascular cancer thrombus |  |  |  |  |
| No | 1.0(reference) |  |  |  |
| Yes | 1.11 (0.40, 3.05) | 0.846 |  |  |
| Bronchial stump |  |  |  |  |
| No | 1.0(reference) |  |  |  |
| Yes | 1.16 (0.53, 2.53) | 0.717 |  |  |
| Pleural invasion |  |  |  |  |
| No | 1.0(reference) |  |  |  |
| Yes | 1.10 (0.65, 1.87) | 0.715 |  |  |
| AJCC^1^ 8th ed. stage |  |  |  |  |
| AJCC<=II stage | 1.0(reference) |  |  |  |
| AJCC>II stage | 1.43 (0.87, 2.33) | 0.156 |  |  |
| Adjuvant chemotherapy |  |  |  |  |
| No | 1.0(reference) |  |  |  |
| Yes | 0.71 (0.34, 1.48) | 0.355 |  |  |
| Adjuvant radiation therapy |  |  |  |  |
| No | 1.0(reference) |  |  |  |
| Yes | 1.14 (0.70, 1.86) | 0.606 |  |  |
| Postoperative targeted therapy |  |  |  |  |
| No | 1.0(reference) |  |  |  |
| Yes | 0.81 (0.25, 2.58) | 0.719 |  |  |
| Two site |  |  |  |  |
| No | 1.0(reference) |  |  |  |
| Yes | 1.23 (0.63, 2.42) | 0.541 |  |  |
| Three or more recurrence sites |  |  |  |  |
| No | 1.0(reference) |  | 1.0(reference) |  |
| Yes | 2.11 (1.10, 4.05) | 0.026 | 2.00(1.04,3.87) | 0.037 |
| Lung recurrence |  |  |  |  |
| No | 1.0(reference) |  |  |  |
| Yes | 0.62 (0.30, 1.30) | 0.208 |  |  |
| Brain recurrence |  |  |  |  |
| No | 1.0(reference) |  |  |  |
| Yes | 2.06 (0.94, 4.54) | 0.072 |  |  |
| Bone recurrence |  |  |  |  |
| No | 1.0(reference) |  |  |  |
| Yes | 0.89 (0.44, 1.81) | 0.757 |  |  |
| Abdominal organs^2^ recurrence |  |  |  |  |
| No | 1.0(reference) |  |  |  |
| Yes | 1.03 (0.47, 2.27) | 0.935 |  |  |
| Pleural recurrence |  |  |  |  |
| No | 1.0(reference) |  |  |  |
| Yes | 0.71 (0.22, 2.26) | 0.561 |  |  |
| Lymph node^4^ recurrence |  |  |  |  |
| No | 1.0(reference) |  |  |  |
| Yes | 0.71 (0.40, 1.26) | 0.238 |  |  |
| Multisite^3^ recurrence |  |  |  |  |
| No | 1.0(reference) |  | 1.0(reference) |  |
| Yes | 1.73 (1.02, 2.91) | 0.040 | 1.21(0.60,2.45) | 0.593 |

Note：1. Abbreviations: BMI, Body Mass Index ; AJCC, American Joint Committee on Cancer ; CI, Confidence interval;HR, Hazard ratio

2. Abdominal organs (liver+Adrenal)

3. Multisite (2 or more organs)

4. Lymph node (hilar, supraclavicular and thoracic lymph nodes)

5. *P* value, using Pearson's Chi-squared test; Wilcoxon rank sum test; Fisher's exact test
